# Grass Carp Reovirus Induces Formation of Lipid Droplets as Sites for Its Replication and Assembly

**DOI:** 10.1128/mbio.02297-22

**Published:** 2022-11-29

**Authors:** Libo He, Qian Wang, Xinyu Liang, Hanyue Wang, Pengfei Chu, Cheng Yang, Yongming Li, Lanjie Liao, Zuoyan Zhu, Yaping Wang

**Affiliations:** a State Key Laboratory of Freshwater Ecology and Biotechnology, Institute of Hydrobiology, Chinese Academy of Sciences, Wuhan, China; b University of Chinese Academy of Sciences, Beijing, China; c College of Animal Science and Technology, Yangzhou University, Yangzhou, China; d Innovative Academy of Seed Design, Chinese Academy of Sciences, Beijing, China; Columbia University Medical College

**Keywords:** grass carp reovirus, lipidomic analysis, lipid droplets, viroplasms, replication, assembly

## Abstract

Grass carp is an important commercial fish in China that is plagued by various diseases, especially the hemorrhagic disease induced by grass carp reovirus (GCRV). Nevertheless, the mechanism by which GCRV hijacks the host metabolism to complete its life cycle is unclear. In this study, we performed lipidomic analysis of grass carp liver samples collected before and after GCRV infection. GCRV infection altered host lipid metabolism and increased *de novo* fatty acid synthesis. Increased *de novo* fatty acid synthesis induced accumulation of lipid droplets (LDs). LDs are associated with GCRV viroplasms, as well as viral proteins and double-stranded RNA. Pharmacological inhibition of LD formation led to the disappearance of viroplasms, accompanied by decreased viral replication capacity. Moreover, transmission electron microscopy revealed LDs in close association with the viroplasms and mounted GCRV particles. Collectively, these data suggest that LDs are essential for viroplasm formation and are sites for GCRV replication and assembly. Our results revealed the detailed molecular events of GCRV hijacking host lipid metabolism to benefit its replication and assembly, which may provide new perspective for the prevention and control of GCRV.

## INTRODUCTION

The grass carp (Ctenopharyngodon idellus), an important aquaculture fish in China, accounts for more than 21% of the total freshwater aquaculture production in the country. Production of grass carp reached 5.57 million tons in 2020, implying the great commercial value of this fish (1). However, grass carp aquaculture has been plagued with many diseases, especially grass carp hemorrhagic disease caused by grass carp reovirus (GCRV), the most virulent pathogen in the genus *Aquareovirus* (2, 3). Consequently, GCRV has received much attention from many researchers who aim to achieve disease-resistant breeding or uncover the mechanisms underlying GCRV infection (4–8). The genome of GCRV consists of 11 double-stranded RNA (dsRNA) segments that encode seven structural proteins (VP1 to VP7) and five nonstructural proteins (NS80, NS38, NS31, NS26, and NS16) (9, 10). Replication and assembly of GCRV occur in cytoplasmic inclusion bodies termed “viroplasms” (11, 12), which contain host and viral components essential for viral morphogenesis (13). As obligate intracellular parasites, viruses are completely dependent on the host metabolism to provide energy and macromolecules to synthesize viral components and for successful replication and assembly (14, 15). Nevertheless, the mechanism by which GCRV hijacks the host metabolism to benefit viral replication and assembly remains unclear. Understanding how GCRV interacts with and manipulates the host cell metabolism will provide important information about the pathogenesis of GCRV infection, as well as potential targets for the prevention and control of this virus.

Lipids, carbohydrates, proteins, and nucleic acids are the four major classes of biological macromolecules fundamental to living organisms (16). Lipids not only are important components of cells and internal organelle membranes but also play crucial roles in various life processes (17). Moreover, lipids participate in various stages of viral infection, including entry, replication, assembly, and energy supply (18). Specific classes of lipids are abundant in the viral envelope structure and are essential for their infectivity (19, 20). Some nonenveloped viruses may utilize lipids as receptors or cofactors for viral entry (21). Host lipid metabolism is altered by viral infection to provide favorable conditions for replication (22). Recent evidence suggests that abnormal lipid metabolism is a crucial factor for the occurrence of many viral infectious diseases (23, 24).

Lipid droplets (LDs) are intracellular structures that store neutral lipids (25). The core components of LDs are triglycerides and cholesterol esters, whereas the surface of LDs consist of a monolayer of phospholipids (26). LDs were traditionally viewed as passive storage depots for excess neutral lipids; however, increasing evidence suggests that LDs are dynamic organelles that are actively involved in diverse cellular processes, such as lipid homeostasis, membrane trafficking, and signal transduction (27). Moreover, many studies have demonstrated that LDs play an important role in the life cycle of various viruses (28). Rabies virus can utilize LDs as a carrier for transport to the cell membrane, resulting in enhanced viral budding (29). LDs are crucial for the assembly of infectious hepatitis C virus (HCV) virions (30). LDs fuel severe acute respiratory coronavirus 2 (SARS-CoV-2) replication and produce dysregulated inflammatory responses (31). However, the specific roles of LDs during reovirus infection, especially during the life cycle of the *Aquareovirus*, remain unknown.

In this study, we performed lipidomic analysis of liver samples collected from grass carp, before and after GCRV infection. We demonstrated that the major effect of GCRV infection is modulation of cellular lipid metabolism, resulting in increased *de novo* fatty acid synthesis. The increased *de novo* fatty acid synthesis led to the accumulation of LDs in fish cells. LDs are sites for GCRV replication and assembly. Our results revealed the detailed molecular events of GCRV hijacking of host lipid metabolism to allow its replication and assembly.

## RESULTS

### GCRV infection altered host lipid metabolism.

**FIG 1:**
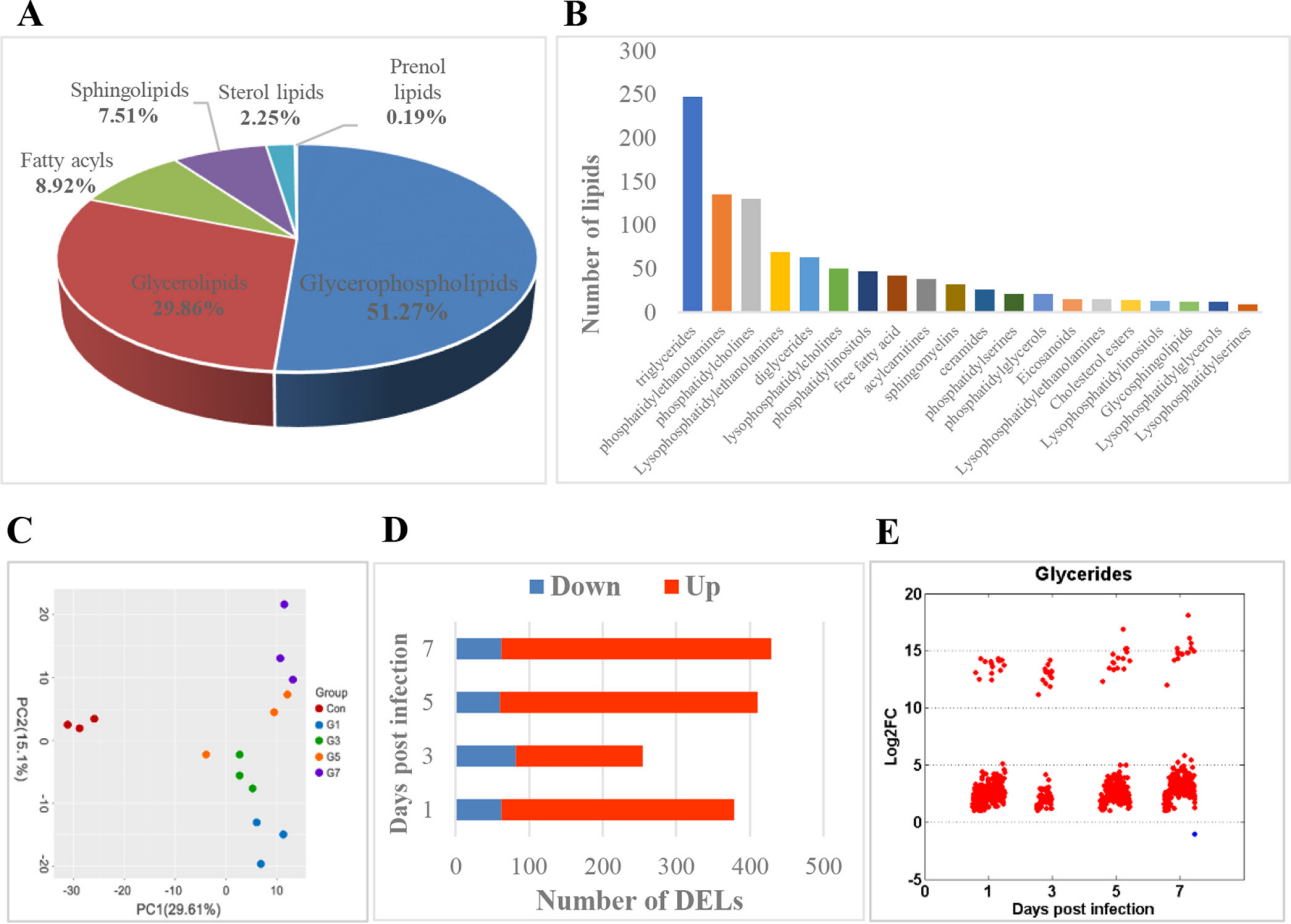
GCRV infection altered host lipid metabolism. (A) Major classes of lipids that identified in all the samples by lipidome analysis. (B) Top 20 subclasses of all identified lipids. (C) PCA score plots for the samples from different groups. (D) Numbers of upregulated and downregulated DELs at different time points after GCRV infection. (E) Expression patterns of lipids belonging to GLs. The red dots represent upregulated DELs, while the blue dots represent downregulated DELs.

To obtain global lipidomic profiles of grass carp after GCRV infection, we performed a widely targeted lipidome analysis on grass carp liver samples collected before (0 day) and at different time points after (1, 3, 5, and 7 days postinfection [dpi]) GCRV infection. Three duplicates were analyzed for samples collected at each time point. A total of 1,065 lipids were identified in all samples (see Table S1 in the supplemental material). The majority of lipids can be divided into several major classes, including glycerophospholipids (GPs), glycerolipids (GLs), fatty acyls (FAs), sphingolipids (SPs), sterol lipids (STs), and prenol lipids (PRs) (Fig. 1A). At the subclass level, triglycerides (TGs) were the most abundant lipids, followed by phosphatidylethanolamines (PEs), phosphocholines (PCs), lysophosphatidylethanolamines (LPEs), diglycerides (DGs), and lysophosphatidylcholines (LPCs) (Fig. 1B). The two score plots of the principal-component analysis (PCA) model show a clear separation of samples before and after GCRV infection, suggesting the effects of GCRV infection (Fig. 1C). Nevertheless, the samples collected at different time points after GCRV infection could not be distinctly distinguished from each other. Orthogonal partial least-squares discrimination analysis (OPLS-DA) was used to identify differentially expressed lipids (DELs). The results showed that 316, 172, 350, and 366 lipids were upregulated, whereas 63, 82, 60, and 63 lipids were downregulated, at 1, 3, 5, and 7 dpi, respectively (Fig. 1D). Furthermore, we also investigated the expression patterns of DELs belonging to five lipid categories: GLs, GPs, FAs, SPs, and STs; the results showed that most DELs in five lipid categories were still upregulated during the GCRV infection process (Fig. 1E and Fig. S1A to D). Obviously, it could be seen that most of lipids were upregulated after GCRV infection regardless of time points, implying that GCRV infection altered host lipid metabolism.

10.1128/mbio.02297-22.1 FIG S1 Expression patterns of lipids belonging to GPs (A), FAs (B), SPs (C), and STs (D). The red dots represent upregulated DELs, while the blue dots represent downregulated DELs. Download FIG S1, TIF file, 1.2 MB. Copyright © 2022 He et al. 2022 He et al. https://creativecommons.org/licenses/by/4.0/ This content is distributed under the terms of the Creative Commons Attribution 4.0 International license.

10.1128/mbio.02297-22.8 TABLE S1 Detailed information on the 1,065 lipids that were identified from all the samples. Download Table S1, XLSX file, 0.3 MB. Copyright © 2022 He et al. 2022 He et al. https://creativecommons.org/licenses/by/4.0/ This content is distributed under the terms of the Creative Commons Attribution 4.0 International license.

### Fatty acid synthesis is essential for GCRV replication.

**FIG 2:**
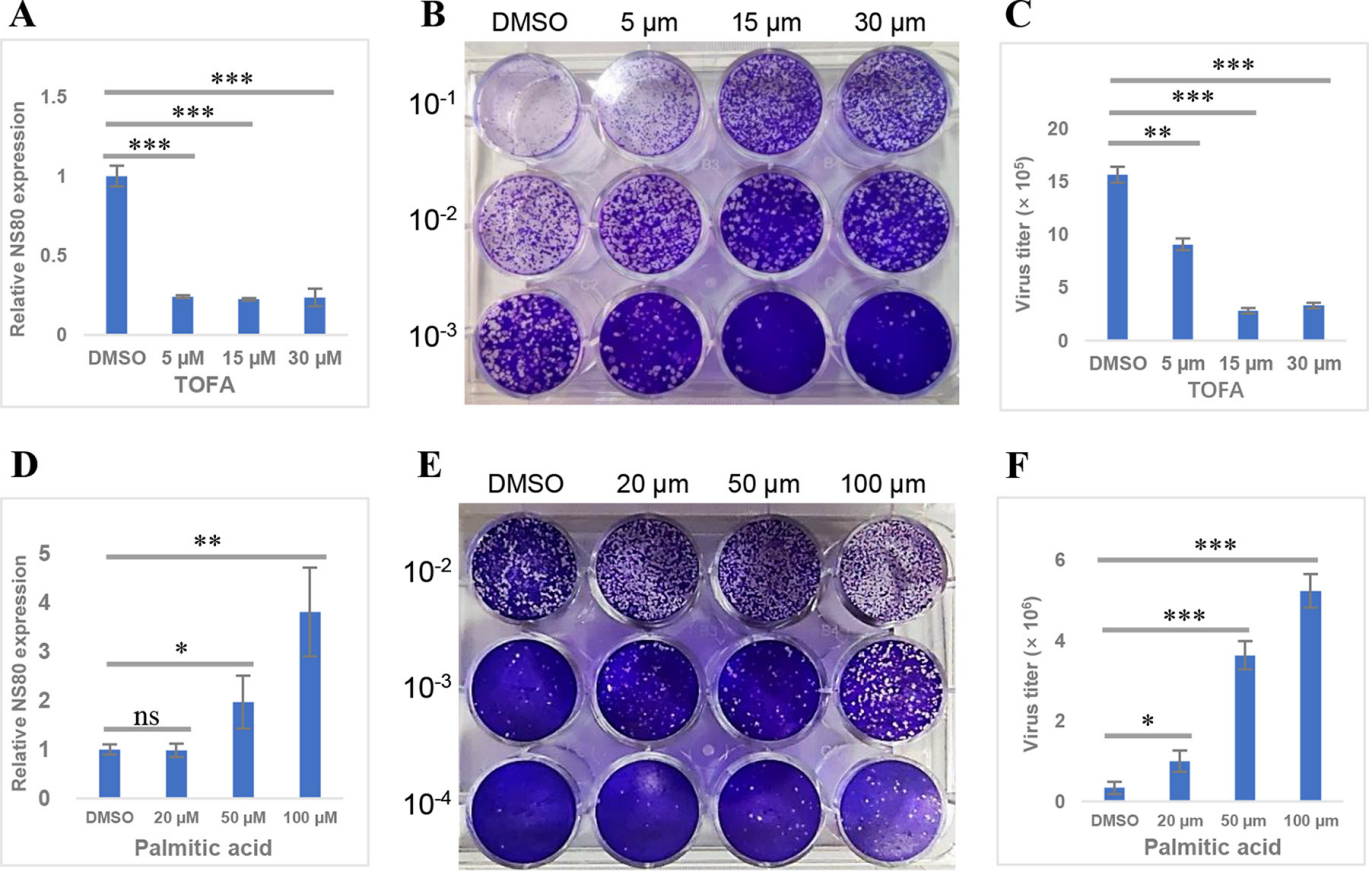
Fatty acid synthesis is essential for GCRV replication. (A and D) Relative gene expression level of *NS80* in cells treated with different concentrations of TOFA or palmitic acid (PA). (B and E) Plaque assay of cells treated with different concentrations of TOFA or PA. (C and F) Virus titers in cells treated with different concentrations of TOFA or PA. ns, no significant difference.

Lipidome analysis revealed that GCRV infection caused the upregulation of most lipids, indicating increased *de novo* fatty acid synthesis. Nevertheless, the specific role of *de novo* fatty acid synthesis in GCRV replication remains unclear. Fatty acid synthase (FASN) enzyme complex and acetyl coenzyme A (acetyl-CoA) carboxylase 1 (ACC1) are pivotal enzymes involved in *de novo* fatty acid synthesis (32). Therefore, we assessed whether *de novo* fatty acid synthesis is critical for GCRV infection by blocking the enzymatic activity of ACC1 and FASN. Grass carp ovary (GCO) cells were treated with compounds C75 and 5-tetradecyloxy-2-furoic acid (TOFA), respective inhibitors of FASN and ACC1, and their effects on viral replication were assessed. The transcripts of viral genes (*NS80* and *VP5*) and viral titers dramatically decreased after treatment with TOFA (Fig. 2A to C and Fig. S2A), whereas the effect of C75 on GCRV replication was not significant (Fig. S2B to D). Furthermore, viral replication was evaluated through the addition of exogenous palmitic acid (PA), a precursor molecule for the synthesis of long-chain fatty acids, phospholipids, and triglycerides (33), to increase *de novo* fatty acid synthesis. The addition of PA to GCRV-infected cells significantly increased viral gene (*NS80* and *VP5*) transcription as well as viral titers compared with those in dimethyl sulfoxide (DMSO)-treated cells (Fig. 2D to F and Fig. S2E). Cell viability detection indicated that these compounds (C75, TOFA, and PA) had no toxic effect on cells at their corresponding concentrations (Fig. S3). Thus, *de novo* fatty acid synthesis was speculated to be essential for GCRV replication.

10.1128/mbio.02297-22.2 FIG S2 Effects of different compounds on GCRV replication. (A) Relative gene expression level of *VP5* in cells treated with different concentrations of TOFA. (B to D) Relative gene expression level of *NS80* (B) and *VP5* (C) and plaque assay (D) of cells treated with different concentrations of C75. (E to G) Relative gene expression level of VP5 in cells treated with different concentrations of palmitic acid (E), atglistatin (F), and CAY10499 (G). (H) Calculated sizes of LDs/viroplasms in GCRV-infected cells harvested at different time points. ns, no significant difference. *, *P* = 0.05 to 0.01; **, *P* = 0.01 to 0.001; ***, *P* ≤ 0.001. Download FIG S2, TIF file, 2.5 MB. Copyright © 2022 He et al. 2022 He et al. https://creativecommons.org/licenses/by/4.0/ This content is distributed under the terms of the Creative Commons Attribution 4.0 International license.

10.1128/mbio.02297-22.3 FIG S3 CCK-8 analysis of the cytotoxicities of different compounds. GCO cells were treated TOFA (A), C75 (B), PA (C), atglistatin (D), CAY10499 (E), triacsin C (F), and IBMX plus isoproterenol (G) at different concentrations for 24 h, and then cell viability was detected by CCK-8 assay. Download FIG S3, TIF file, 1.1 MB. Copyright © 2022 He et al. 2022 He et al. https://creativecommons.org/licenses/by/4.0/ This content is distributed under the terms of the Creative Commons Attribution 4.0 International license.

### Triglycerides are the significant upregulated lipids after GCRV infection.

**FIG 3:**
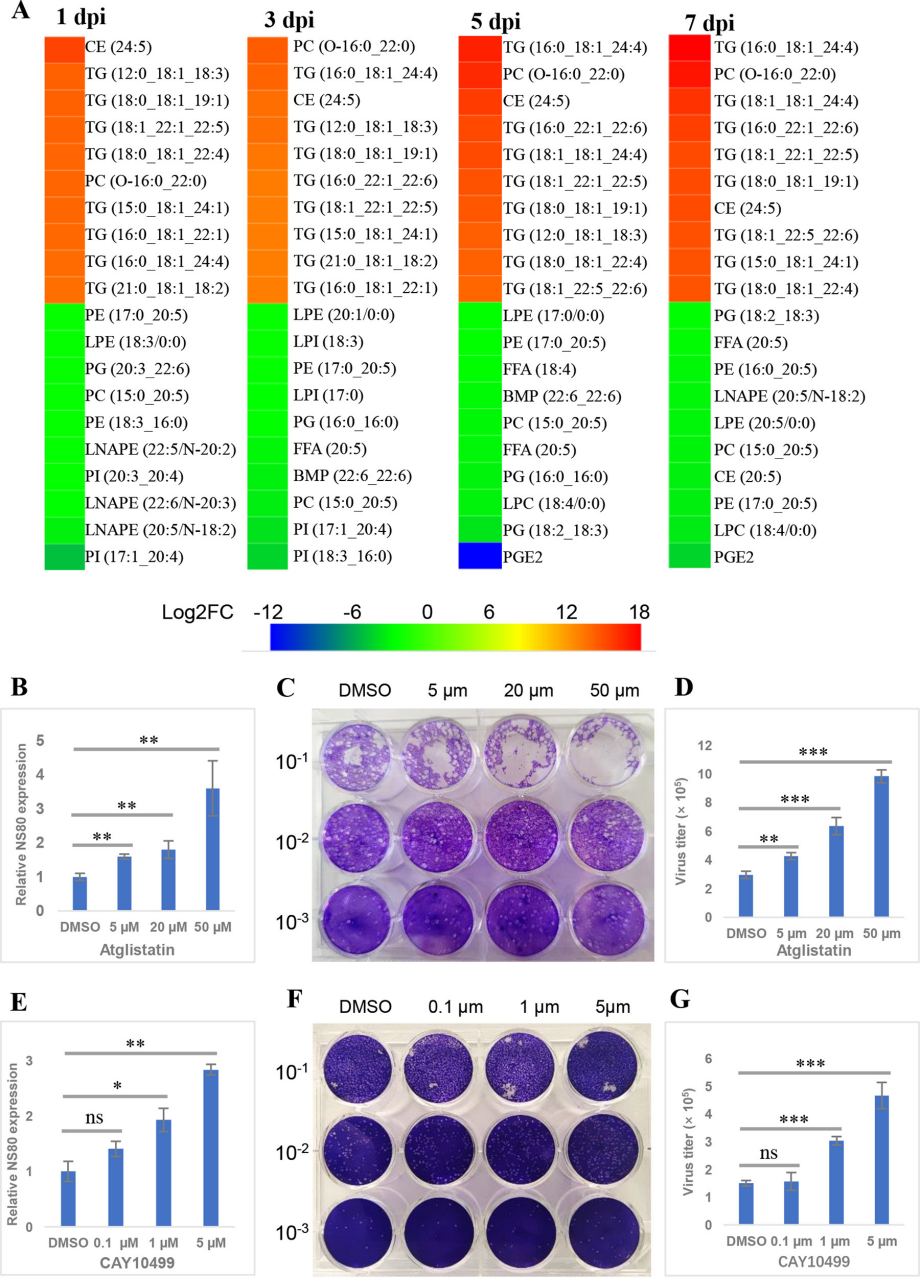
Triglycerides enhanced GCRV replication. (A) Heat map of the top 10 upregulated and downregulated DELs at different time points after GCRV infection. (B and E) Relative gene expression level of *NS80* in cells treated with different concentrations of atglistatin or CAY10499. (C and F) Plaque assay of cells treated with different concentrations of atglistatin or CAY10499. (D and G) Virus titers in cells treated with different concentrations of atglistatin or CAY10499.

It is generally recognized that more significant DELs may play an important role in the response to stimulation; therefore, the top 10 significant DELs (both upregulated and downregulated) after GCRV infection were identified. As shown in Fig. 3A, most of the top 10 upregulated DELs were TGs at all time points and all of them had log_2_ fold changes (FC) of >10, indicating the dramatic upregulation of these TGs. However, almost all of the top 10 downregulated DELs were GPs, regardless of time points, such as PEs, phosphatidylcholines (PCs), phosphatidylinositols (PIs), phosphatidylglycerols (PGs), LPCs, and LPEs. TGs are processed by lipases such as adipose triglyceride lipase (ATGL) and hormone-sensitive lipase (HSL), resulting in the release of fatty acids (34). To further investigate the role of TGs in GCRV infection, GCO cells were treated with atglistatin and CAY10499, respective inhibitors of ATGL and HSL, and their effects on viral replication were assessed. As shown in Fig. 3B to G and Fig. S2F and G, atglistatin and CAY10499 enhanced viral gene (*NS80* and *VP5*) transcription and viral titers in a dose-dependent manner, compared with those in DMSO-treated cells, whereas neither had any toxic effect on cells at the examined concentrations (Fig. S3). Collectively, these data suggest that TGs are significant upregulated lipids after GCRV infection and that TGs could enhance GCRV replication.

### GCRV infection induced formation of lipid droplets.

**FIG 4:**
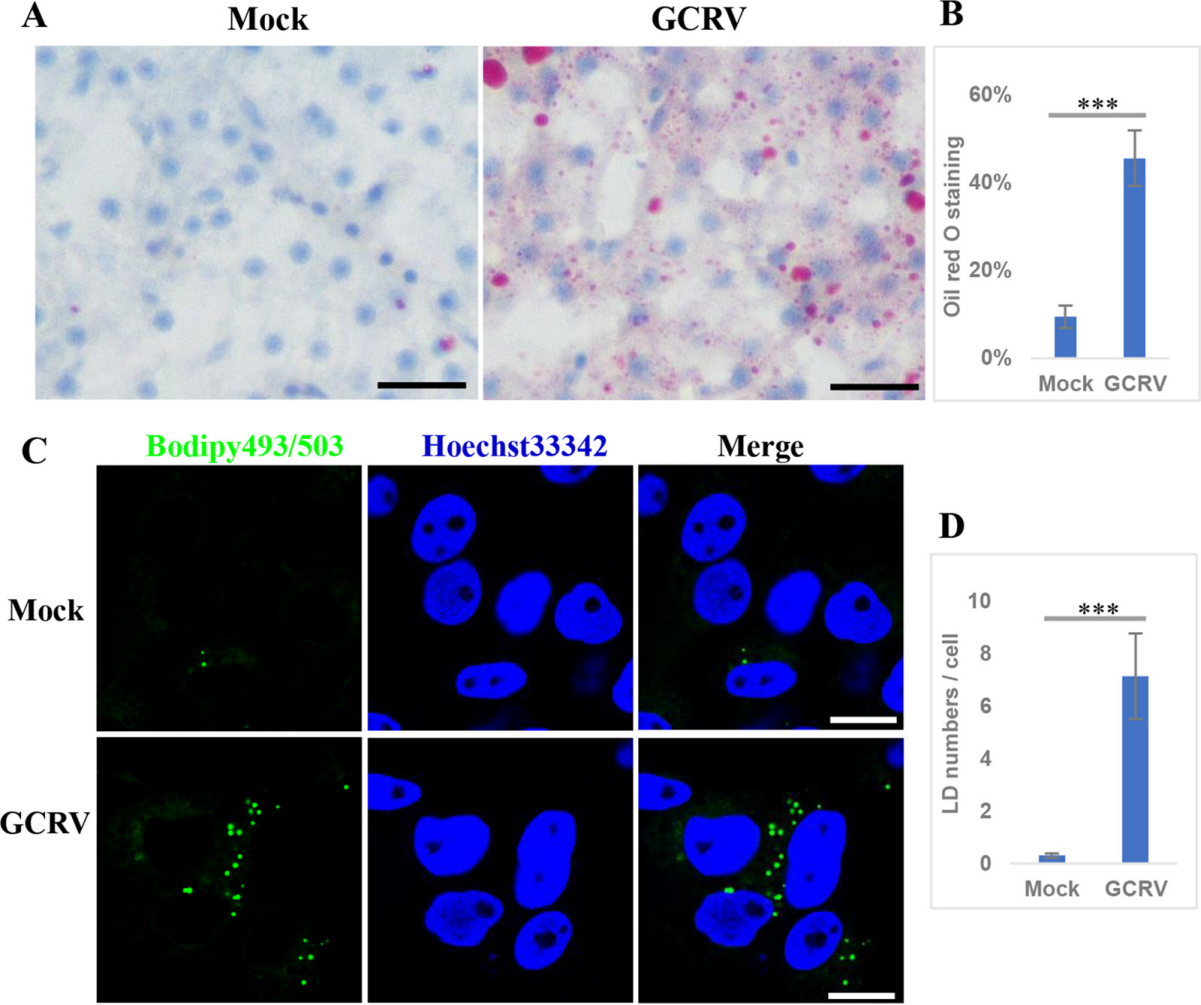
GCRV infection induced formation of lipid droplets. (A) Oil red O staining of liver samples collected from GCRV-infected and uninfected fish. Scale bar = 50 μm. (B) Calculated oil red O staining areas in GCRV-infected and uninfected samples. (C) Bodipy 493/503 staining of mock-infected or GCRV-infected cells. Scale bar = 10 μm. (D) Calculated LD numbers/cell in mock-infected or GCRV-infected cells.

LDs are intracellular structures that store neutral lipids such as TGs, cholesterol esters, and phospholipids (28). Aforementioned results suggest that most lipids, especially TGs, were upregulated after GCRV infection; therefore, we investigated whether these upregulated lipids contributed to LD formation. Grass carp were mock infected or infected with GCRV, and liver samples were collected and stained with oil red O to visualize LDs. As shown in Fig. 4A and B, many cells in the GCRV-infected sample were stained red, whereas only a few cells were stained with oil red O in the mock-infected sample, implying the formation of LDs after GCRV infection. Moreover, we analyzed whether GCRV infection induced LD formation *in vitro*. GCO cells were mock infected or infected with GCRV and stained with Bodipy 493/503, a fluorescent dye specific for LDs. Figure 4C and D show that several LDs, presented as globular or punctiform structures, were detected in GCRV-infected cells, whereas the LDs were difficult to detect in mock-infected cells. Collectively, these results suggest that GCRV infection induces LD formation.

### Lipid droplets associate with GCRV viroplasms.

**FIG 5:**
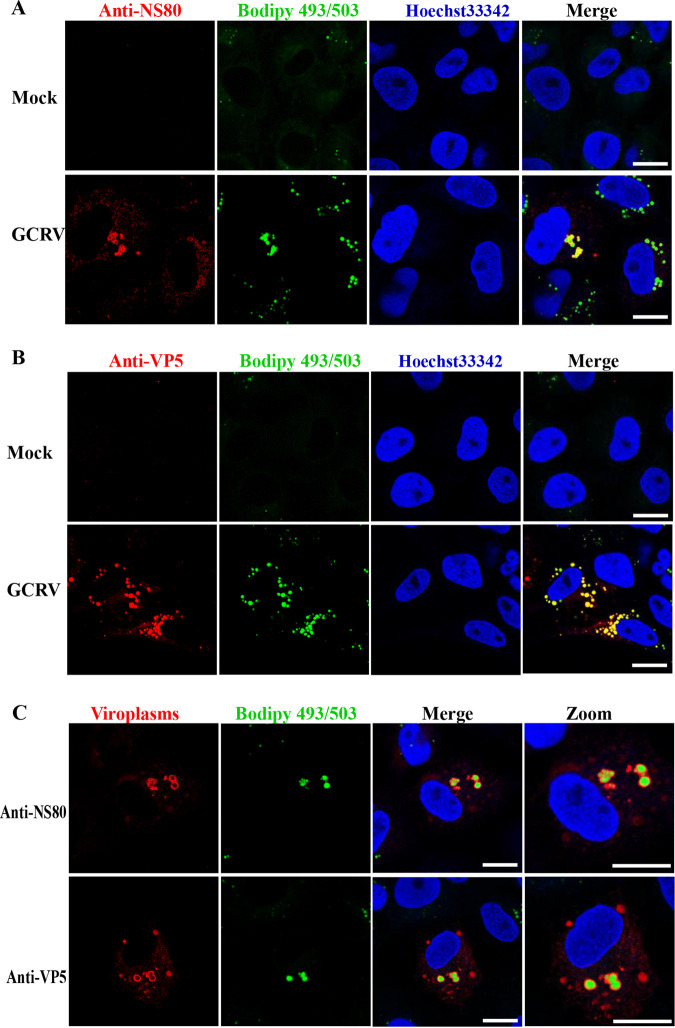
Lipid droplets associate with GCRV viroplasms. (A and B) Confocal microscopy analysis of the relationship between LDs and viroplasms stained with anti-NS80 (A) or anti-VP5 (B). Cells were mock infected or infected with GCRV, and then LDs and viroplasms were stained with Bodipy 493/503 and anti-NS80 antibody (A) and anti-VP5 (B) antibody, respectively. Scale bar = 10 μm. (C) The precise relationship between LDs and viroplasms. Scale bar = 10 μm.

Members of the family *Reoviridae* are known to replicate and assemble in cytoplasmic inclusion bodies termed viroplasms (11, 35, 36). The morphology of LDs (Fig. 4C) was similar to that of the viroplasms formed by GCRV, which prompted us to investigate their relationship. GCO cells were mock infected or infected with GCRV, and LDs and viroplasms were stained with Bodipy 493/503 and anti-NS80 and anti-VP5 antibodies, respectively. As shown in Fig. 5A, the viroplasms stained with anti-NS80 antibody presented as globular inclusion structures, which displayed obvious colocalization with LDs stained with Bodipy 493/503. Identical results were obtained when viroplasms were stained with the anti-VP5 antibody (Fig. 5B). The relationship between LDs and viroplasms was further investigated. In some GCRV-infected cells, confocal microscopy revealed that viroplasms stained with anti-NS80 or anti-VP5 antibodies, particularly the larger viroplasms, appeared as ring-shaped structures around LDs (Fig. 5C). Inspected together, our data indicate that LDs are associated with GCRV viroplasms and may benefit viroplasm formation.

**FIG 6:**
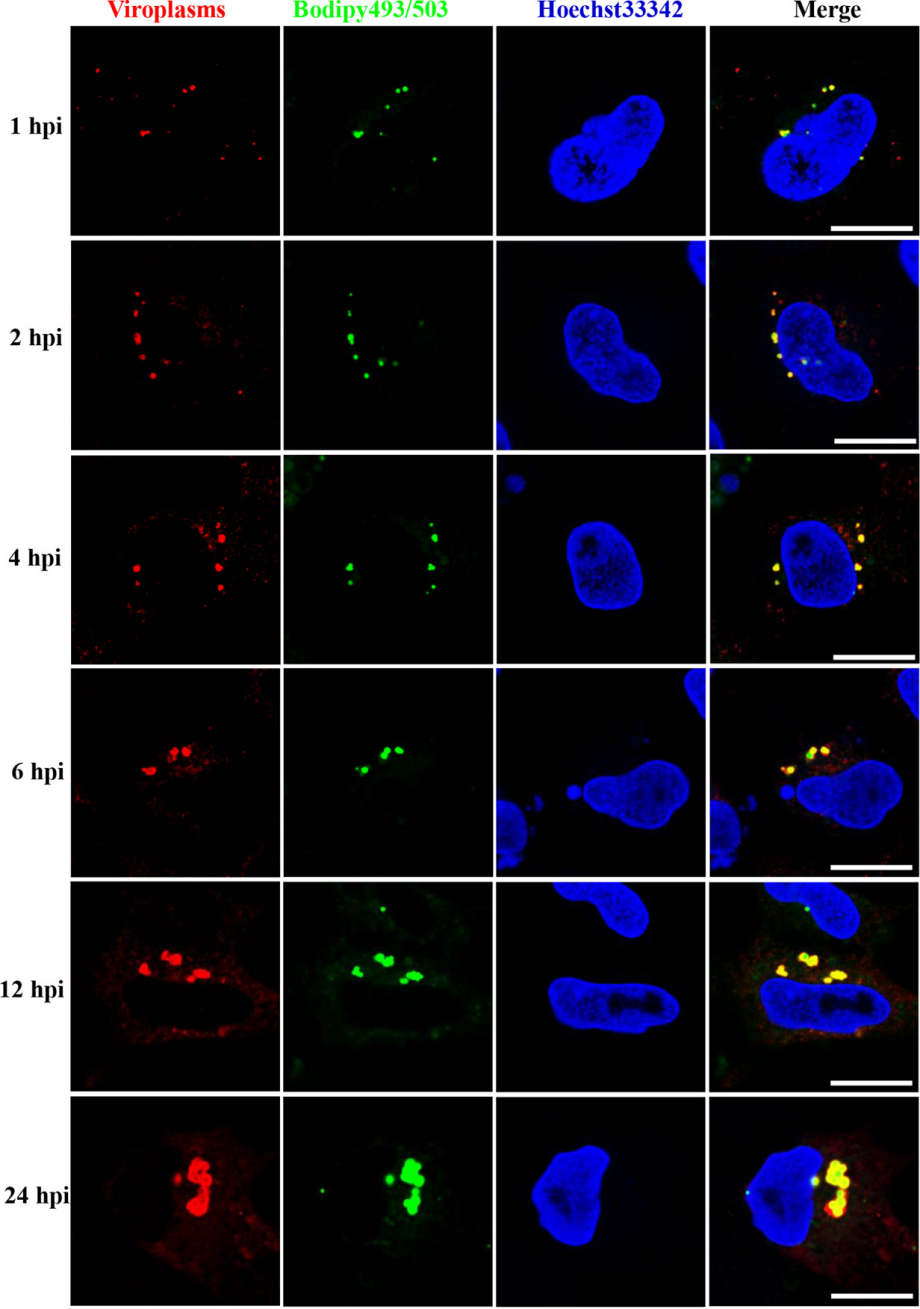
The relationship between LDs and viroplasms at different time points after GCRV infection. GCO cells were infected with GCRV and harvested at 1, 2, 4, 6, 12, and 24 hpi. Viroplasms and LDs were stained with anti-NS80 antibody and Bodipy 493/503, respectively. Scale bar = 10 μm.

To further determine whether LD formation occurred prior to or concomitant with viroplasms, we performed a time course experiment to investigate the relationship between LDs and viroplasms. GCO cells were infected with GCRV at a multiplicity of infection (MOI) of 1 and harvested at 1, 2, 4, 6, 12, and 24 h postinfection (hpi). Viroplasms and LDs were stained with anti-NS80 antibody and Bodipy 493/503, respectively. As shown in Fig. 6, small puncta of LDs and viroplasms were detected in GCRV-infected cells as early as 1 hpi, and most LDs colocalized with viroplasms. As the infection progressed, the size of LDs and viroplasms increased (Fig. 6 and Fig. S2H), and the colocalization between LDs and viroplasms became more obvious. Therefore, these results indicate that LD formation is concomitant with viroplasm assembly.

### Lipid droplets are essential for viroplasm formation and GCRV replication.

**FIG 7:**
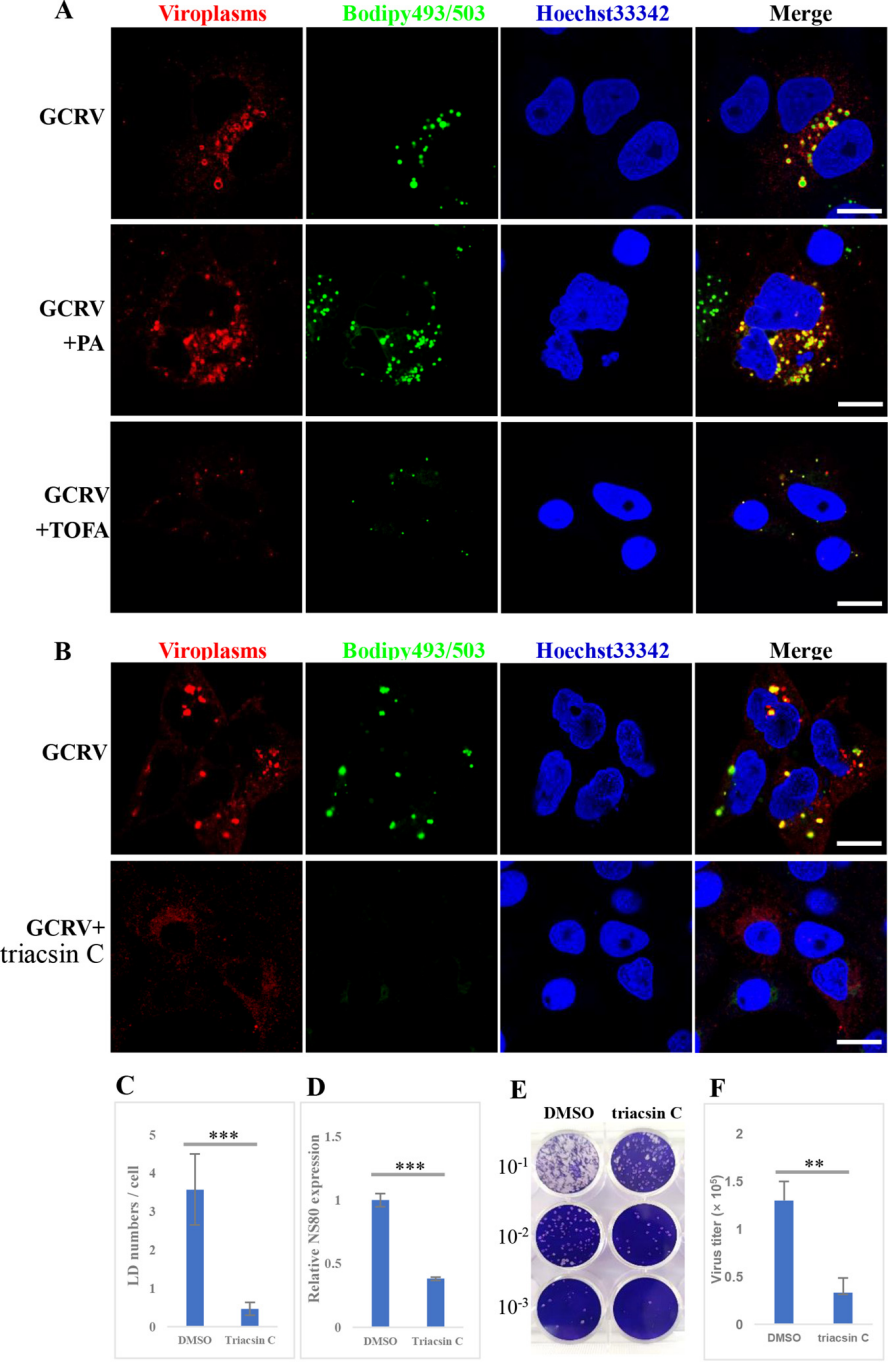
Lipid droplets are essential for viroplasm formation and GCRV replication. (A) Viroplasms and LDs in GCRV-infected cells after treatment with PA or TOFA. GCO cells were infected with GCRV and then treated with PA or TOFA. The LDs and viroplasms were stained with Bodipy 493/503 and anti-NS80 antibody, respectively. Scale bar = 10 μm. (B) The viroplasms and LDs in GCRV-infected cells after treatment with triacsin C. GCO cells were infected with GCRV and then treated with triacsin C. The LDs and viroplasms were stained as described above. Scale bar = 10 μm. (C) Calculated LD numbers/cell in GCRV-infected cells after treatment or not with triacsin C. (D) Relative gene expression level of *NS80* in GCRV-infected cells after treatment or not with triacsin C. (E) Plaque assay of GCRV-infected cells after treatment or not with triacsin C. (F) Virus titers in GCRV-infected cells after treatment or not with triacsin C.

Our results show that *de novo* fatty acid synthesis is essential for GCRV replication (Fig. 2). *De novo* fatty acid synthesis induces the accumulation of TGs and formation of LDs. Therefore, we investigated whether the effects of *de novo* fatty acid synthesis on GCRV replication depended on LD formation. GCO cells were infected with GCRV and treated with PA (50 μM) or TOFA (15 μM) to enhance or inhibit *de novo* fatty acid synthesis. LDs and viroplasms were stained with Bodipy 493/503 and anti-NS80 antibody, respectively. As expected, GCRV infection caused the formation of LDs that colocalized with viroplasms (Fig. 7A). Moreover, PA treatment significantly increased the number of LDs and viroplasms in GCRV-infected cells (Fig. 7A and Fig. S4A), and colocalization between LDs and viroplasms was observed. Nevertheless, the number and size of LDs, as well as viroplasms, were significantly reduced when the infected cells were treated with TOFA (Fig. 7A and Fig. S4A and B). These results implied that the effects of *de novo* fatty acid synthesis on GCRV replication may depend on LD formation.

10.1128/mbio.02297-22.4 FIG S4 Lipid droplets are essential for viroplasm formation and GCRV replication. (A) Calculated LD numbers/cell in GCRV-infected cells after treatment with PA or TOFA. (B) Calculated size of LDs in GCRV-infected cells after treatment with PA or TOFA. (C) Relative gene expression level of *VP5* in GCRV-infected cells after treatment or not with triacsin C. (D) Viroplasms and LDs in GCRV-infected cells after treatment with IBMX and isoproterenol. (E) Calculated size of LDs in GCRV-infected cells after treatment or not with IBMX and isoproterenol. (F to H) Relative gene expression level of *NS80* (F), plaque assay (G), and virus titer (H) in GCRV-infected cells after treatment or not with IBMX and isoproterenol. Download FIG S4, TIF file, 2.3 MB. Copyright © 2022 He et al. 2022 He et al. https://creativecommons.org/licenses/by/4.0/ This content is distributed under the terms of the Creative Commons Attribution 4.0 International license.

To further investigate the important role of LDs in GCRV replication and viroplasm formation, cells were treated with 5 μM triacsin C, a specific acyl-CoA synthetase inhibitor, to block LD formation (37). Figure 7B and C show that LDs were difficult to detect in GCRV-infected cells after treatment with triacsin C. Moreover, the viroplasms stained with anti-NS80 antibody appeared to be uniformly dispersed in the cytoplasm of GCRV-infected cells in the presence of triacsin C. Reverse transcription-quantitative PCR (RT-qPCR) revealed that mRNA expression levels of viral genes (*NS80* and *VP5*) in the triacsin C-treated cells were significantly reduced compared with those in DMSO-treated cells (Fig. 7D and Fig. S4C). The plaque number and viral titer in triacsin C-treated cells were also significantly decreased (Fig. 7E and F). Detection of cell viability indicated that 5 μM triacsin C had no toxic effect on the cells (Fig. S3).

Previous studies have shown that LDs may disperse into smaller microdroplets when treated with Isobutylmethylxanthine (IBMX) and isoproterenol (38). In this study, GCRV-infected cells were treated with 0.5 mM IBMX combined with 10 μM isoproterenol after determining the cell cytotoxicity (Fig. S3) and harvested at 12 hpi. As shown in Fig. S4, after treatment with IBMX and isoproterenol, the sizes of LDs were significantly reduced (Fig. S4D and E). Meanwhile, globular viroplasms were difficult to detect in GCRV-infected cells in the presence of IBMX and isoproterenol (Fig. S4D). The mRNA expression level of the viral gene (*NS80*), plaque number, and viral titer in the drug-treated cells were significantly reduced compared with those of DMSO-treated cells (Fig. S4F to H). Collectively, these results indicated that LDs are essential for viroplasm formation and GCRV replication.

### GCRV viral proteins recruited by LDs.

**FIG 8:**
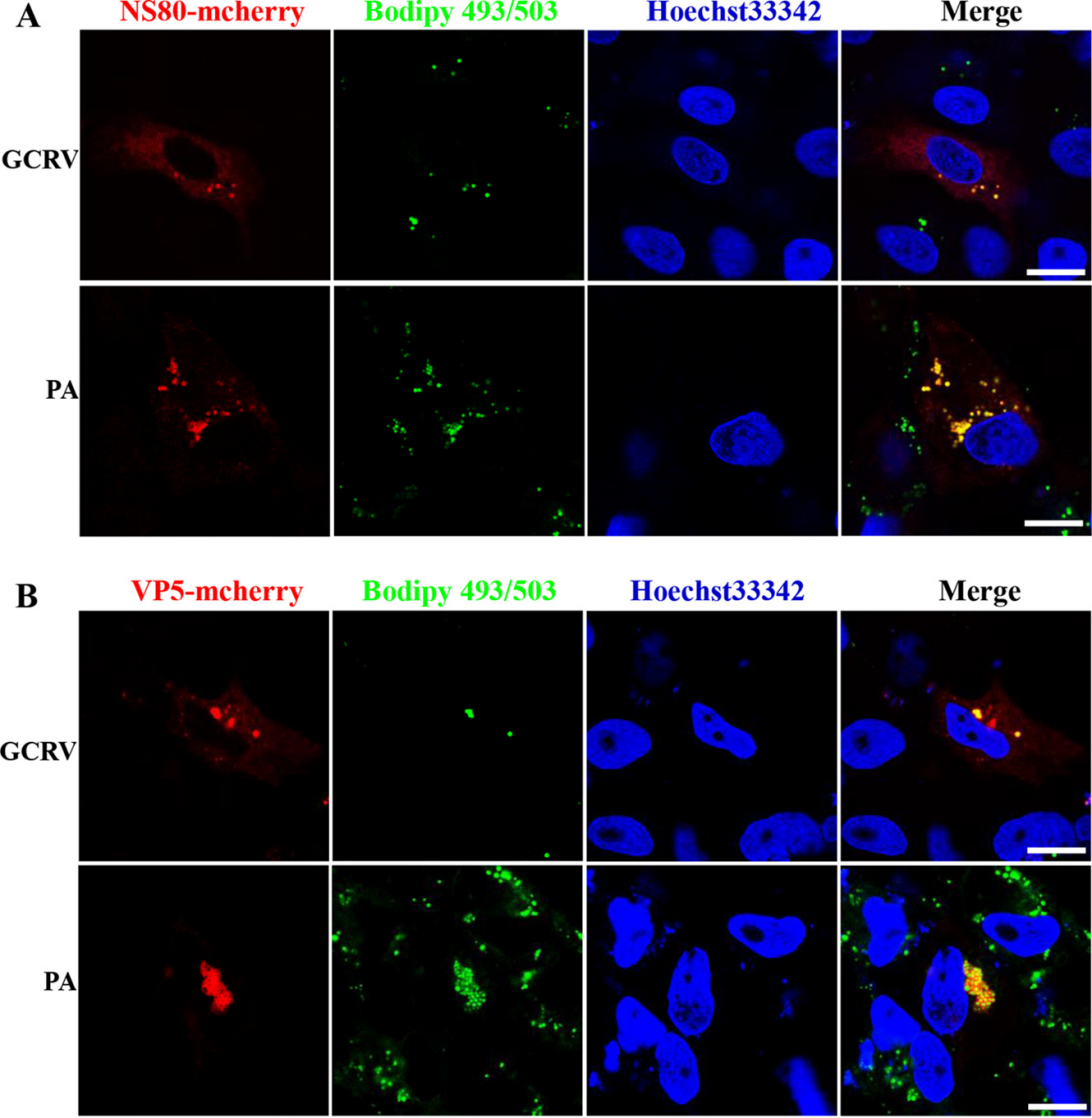
GCRV-encoded proteins recruited by LDs. (A and B) Confocal microscopy analysis of the relationship between LDs and ectopic expression of NS80-mCherry fusion protein (A) or VP5-mCherry fusion protein (B). Cells were transfected with pmCherry-NS80 plasmid (A) or pmCherry-VP5 plasmid (B) and then infected with GCRV or treated with PA to induce LD formation. The relationship between LDs and NS80-mcherry fusion protein (A) or VP5-mcherry fusion protein (B) was analyzed by confocal microscopy. Scale bar = 10 μm.

The viroplasms of reoviruses contain viral proteins that are essential for viral replication and assembly. The association between viroplasms and LDs prompted us to investigate which viral proteins play a pivotal role during this association or are recruited by LDs. A total of 12 proteins are encoded by GCRV (9, 10), of which NS80 is crucial for viroplasm formation (11, 39). Therefore, we investigated the relationship between LDs and NS80. As expected, ectopic expression of the NS80-mCherry fusion protein in transfected cells formed viroplasm-like structures (VLS) (Fig. S5A). In addition, transfected cells were infected with GCRV or treated with PA to induce LD formation. Figure 8A shows obvious colocalization between the NS80-mCherry fusion protein and LDs in GCRV-infected or PA-treated cells. Moreover, in some cases, we observed that the VLS appeared as ring-shaped structures around LDs, particularly the larger VLS (Fig. S6A), indicating that NS80 was recruited by LDs.

10.1128/mbio.02297-22.5 FIG S5 Subcellular localization patterns of 12 mCherry-fusion GCRV proteins and relationship between LDs and 12 mCherry-fusion GCRV proteins. (A) Subcellular localization patterns of 12 mCherry-fusion GCRV proteins in transfected cells. Cells were transfected with plasmids and harvested at 24 h for confocal microscopy observation. Scale bar = 10 μm. (B) Relationship between LDs and 12 mCherry-fused GCRV proteins. Cells were transfected with plasmids and then treated with PA to induce LD formation; then cells were harvested at 24 h for confocal microscopy observation. Scale bar = 10 μm. Download FIG S5, TIF file, 1.8 MB. Copyright © 2022 He et al. 2022 He et al. https://creativecommons.org/licenses/by/4.0/ This content is distributed under the terms of the Creative Commons Attribution 4.0 International license.

10.1128/mbio.02297-22.6 FIG S6 GCRV-encoded proteins recruited by LDs. Confocal microscopy analysis of the relationship between LDs and ectopic expression of NS80-mCherry fusion protein (A) or VP5-mCherry fusion protein (B). Cells were transfected with corresponding plasmids and then infected with GCRV or treated with PA to induce LD formation. The relationship between LDs and NS80-mCherry fusion or VP5-mCherry fusion protein was analyzed by confocal microscopy. Scale bar = 10 μm. Download FIG S6, TIF file, 1.2 MB. Copyright © 2022 He et al. 2022 He et al. https://creativecommons.org/licenses/by/4.0/ This content is distributed under the terms of the Creative Commons Attribution 4.0 International license.

We also investigated the relationship between LDs and other GCRV-encoded viral proteins. As shown in Fig. S5A, ectopic expression of the remaining 11 mCherry-fusion proteins in transfected cells did not form VLS. All of them were distributed uniformly in the cytoplasm of transfected or whole cells. When the transfected cells were infected with GCRV or treated with PA to induce LD formation, we surprisingly found that the VP5-mCherry fusion protein also presented as VLS and colocalized with LDs in GCRV-infected or PA-treated cells (Fig. 8B). More interestingly, we also found some LDs surrounded or packaged by the VLS formed by the VP5-mCherry fusion protein (Fig. S6B), indicating that VP5 was recruited by LDs. However, the remaining GCRV-encoded viral proteins could not be recruited by PA-induced LDs (Fig. S5B).

### LDs are sites for GCRV replication and assembly.

**FIG 9:**
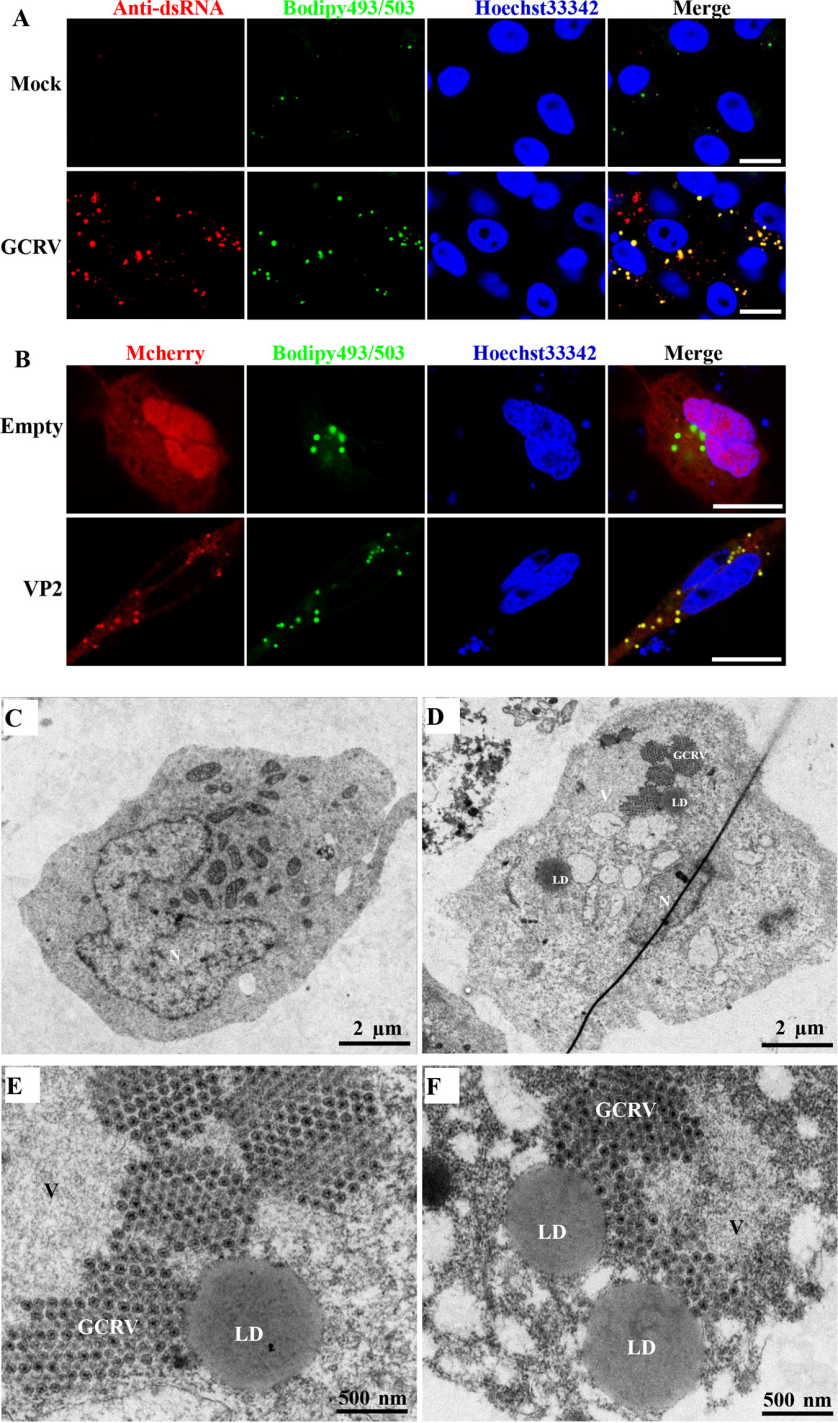
LDs are sites for GCRV replication and assembly. (A) The relationship between LDs and viral RNA. GCO cells were mock infected or infected with GCRV, and then LDs and viral RNA were stained with Bodipy 493/503 and a specific antibody for double-stranded RNA (dsRNA), respectively. Scale bar = 10 μm. (B) Relationship between LDs and VP2 in the presence of GCRV. GCO cells were transfected with VP2-mCherry plasmid or empty mCherry plasmid and then infected with GCRV to induce LD formation. Then cells were harvested at 24 h for confocal microscopy observation. Scale bar = 10 μm. (C and D) Transmission electron microscopy (TEM) analysis of mock-infected (C) or GCRV-infected (D) cells. N, nucleus; V, viroplasms; LDs, lipid droplets; GCRV, mounted GCRV particles. Scale bar = 2 μm. (E and F) Magnification of TEM pictures of GCRV-infected cells. Scale bar = 500 nm.

The viroplasms of reoviruses also contain viral RNA that participates in viral replication and assembly. Therefore, we investigated the relationship between LDs and viral RNA. GCO cells were mock infected or infected with GCRV, and LDs and viral RNA were stained with Bodipy 493/503 and a specific antibody for double-stranded RNA (dsRNA), respectively. As shown in Fig. 9A, strong labeling of dsRNA in GCRV-infected cells was observed compared with that in mock-infected cells. Moreover, we also observed colocalization between viral dsRNA and Bodipy 493/503-stained LDs, implying that LDs may be sites for GCRV replication.

The synthesis of GCRV dsRNA was catalyzed by VP2 protein, which is the RNA-dependent RNA polymerase (RdRp) that is encoded by the S2 segment (10). Hence, we investigated the relationship between VP2 and LDs in the presence of GCRV to further elucidate the role of LDs during GCRV replication. Due to the lack of antibody against VP2, we transfected VP2-mCherry plasmid or empty mCherry plasmid into GCO cells and then infected the cells with GCRV to induce LD formation. Interestingly, obvious colocalization between the VP2-mCherry fusion protein and GCRV-induced LDs was observed (Fig. 9B), indicating that LDs are sites for GCRV dsRNA synthesis. Moreover, we also found colocalization between ectopically expressed VP2 and viroplasms or NS80-EGFP fusion protein (Fig. S7A and B), suggesting that the recruitment of VP2-mCherry by GCRV-induced LDs may be mediated by viroplasms or NS80.

10.1128/mbio.02297-22.7 FIG S7 LDs are sites for GCRV replication and assembly. (A) Relationship between VP2 and viroplasms. GCO cells transfected with VP2-EGFP plasmid or empty EGFP plasmid and infected with GCRV were harvested at 24 h for confocal microscopy observation. Scale bar = 10 μm. (B) Relationship between VP2 and NS80. GCO cells were cotransfected with NS80-EGFP plasmid and VP2-mCherry or empty mCherry plasmid and then harvested at 24 h for confocal microscopy observation. Scale bar = 10 μm. (C) Transmission electron microscopy (TEM) analysis of GCRV-infected cells. N, nucleus; V, viroplasms; LDs, lipid droplets; GCRV, the mounted GCRV particles. Scale bar = 2 μm. (D) Magnification of TEM pictures of GCRV-infected cells. Scale bar = 500 nm. Download FIG S7, TIF file, 1.8 MB. Copyright © 2022 He et al. 2022 He et al. https://creativecommons.org/licenses/by/4.0/ This content is distributed under the terms of the Creative Commons Attribution 4.0 International license.

To further analyze the role of LDs in GCRV replication and assembly, GCO cells were mock infected or infected with GCRV for 48 h and then analyzed using transmission electron microscopy (TEM). Like Bodipy 493/503 staining in GCRV-infected cells, TEM revealed that GCRV infection induced the accumulation of LDs (Fig. 9D and Fig. S7) compared with the case with mock-infected cells (Fig. 9C). Consistent with the data obtained by confocal microscopy, we observed by TEM that the viroplasms and mounted GCRV particles were closely associated with LDs, particularly the fully assembled viral particles, which are associated with the monolayer membranes of LDs (Fig. 9E and F and Fig. S7C and D), indicating that LDs are sites for GCRV assembly.

## DISCUSSION

Viruses are obligate intracellular parasites that require host cell machinery to complete their life cycle (40). Some RNA viruses have been reported to modulate host lipid metabolism and hijack LDs to benefit viral replication and assembly (30, 41, 42), whereas several reports have suggested that LDs are required for an early efficient antiviral interferon response (43, 44). Therefore, the particular role of LDs during viral infection may vary according to the virus and infected host cell. GCRV is a dsRNA virus belonging to the *Aquareovirus* genus of the *Reoviridae* family (2). How GCRV hijacks host cell metabolism and the mechanisms employed by GCRV to support its replication in host cells remain unknown. In this study, we demonstrated that GCRV infection alters host lipid metabolism and causes *de novo* fatty acid synthesis. *De novo* fatty acid synthesis induced accumulation of LDs as sites for GCRV replication and assembly. Our results illustrate a typical example of how the virus hijacks cellular organelles for replication and assembly.

Previously, LDs were considered simple storage structures; however, recent findings indicate that LDs are dynamic organelles involved in diverse biological processes, particularly during pathogen infection (28). Bodipy 493/503 staining showed that many LDs presented as globular or punctiform structures in GCRV-infected cells (Fig. 4C), which is similar to the case with the viroplasms formed by GCRV infection. Viroplasms are cytoplasmic inclusion bodies for viral morphogenesis and viral RNA replication in reovirus-infected cells (11, 36); the underlying mechanisms of viroplasm occurrence remain largely unknown. We showed that GCRV-induced LDs colocalized with viroplasms, which occurred concomitantly with viroplasm assembly as early as 1 hpi, and that pharmacological inhibition of LD formation led to the disappearance or morphological change of viroplasms accompanied by reduced virus replication capacity. Moreover, further study revealed that viral proteins, viral dsRNA, viral RdRp, and mounted viral particles colocalized or were closely associated with LDs. These results imply that the occurrence of viroplasms is dependent on LD formation; hence, LDs are the sites or scaffolds for GCRV replication and assembly.

Some reovirus-encoded proteins are critical for the viroplasm formation (45). In GCRV, the nonstructural protein NS80 is crucial for recruiting viral components to form viroplasms (11, 12). Fluorescence observations showed that LDs colocalized with viroplasms that were stained with anti-NS80 antibody, as well as the VLS formed by ectopic expression of the NS80-mCherry fusion. These data indicate that the association between LDs and viroplasms may be mediated by NS80. Viroplasms contain viral components, such as viral RNA and proteins, that are required for virus replication and assembly. Therefore, we investigated the relationship between LDs and other ectopically expressed viral proteins. Surprisingly, ectopic expression of the VP5-mCherry fusion protein was distributed uniformly in transfected cells in the absence of LDs, whereas VLS were formed in the presence of LDs and colocalized with LDs, implying that VP5 was also recruited by LDs. VP5 is the outer capsid protein of GCRV and may be necessary for GCRV cell attachment or receptor binding (46, 47). TEM showed that not only the viroplasms but also the mounted GCRV particles were closely associated with LDs. Therefore, it could be proposed that NS80 is responsible for the association between LDs and viroplasms, while VP5 is involved in the association between LDs and mounted viral particles.

The surfaces of LDs contain many proteins, including perilipin, adipophilin, and various other cellular proteins (48). However, it remains unclear which LD proteins are crucial for the association between LDs and viroplasms or mounted viral particles. The identification of these LD proteins will provide not only insights into the mechanism of viroplasm morphogenesis but also a potential genetic target for breeding of disease-resistant grass carp or prevention and control of GCRV. Moreover, an increasing number of reports indicate that many pathogens are dependent on interactions with LDs for their replication (28), including viruses (hepatitis C virus, dengue virus, rabies virus, rotavirus, etc.), bacteria (Mycobacterium tuberculosis, Mycobacterium bovis, Mycobacterium leprae, and Chlamydia trachomatis), and parasites (Trypanosoma cruzi, Leishmania amazonensis, Leishmania major, and Toxoplasma gondii). The role of LDs during host-pathogen interactions is now emerging as a research topic (30, 49); therefore, the development of compounds that perturb LD homeostasis or blocked the interaction between LDs and pathogens may have the potential to become broad-spectrum antimicrobial drugs.

In conclusion, we performed lipidomic analysis of liver samples collected before and after GCRV infection in grass carp and demonstrated that the major effect of GCRV infection are modulation of cellular lipid metabolism, which results in *de novo* fatty acid synthesis. *De novo* fatty acid synthesis induced the accumulation of LDs. LDs are associated with GCRV viroplasms and are sites of GCRV replication and assembly. Our results revealed the detailed molecular events of GCRV hijacking of host lipid metabolism to allow its replication and assembly and may provide new insights for the prevention and control of GCRV.

## MATERIALS AND METHODS

### Cell, virus, antibodies, and reagents.

GCO cells were cultured in M199 medium (HyClone, USA) supplemented with 10% fetal bovine serum (FBS), 100 IU/mL of penicillin, and 100 mg/mL of streptomycin under humidified conditions with 5% CO_2_ at 28°C. Grass carp reovirus, which was isolated and identified as subtype I by our laboratory (50), was used in the study of viral infection. Rabbit polyclonal antibodies against the GCRV nonstructural protein NS80 (anti-NS80) and structural protein VP5 (anti-VP5) were prepared in our laboratory (51). Mouse anti-double-stranded RNA (J2 clone) antibody was purchased from Nordic-MUbio (Susteren, Netherlands). Horseradish peroxidase (HRP)-conjugated goat anti-rabbit IgG was purchased from Sigma-Aldrich (St. Louis, MO, USA). Alexa Fluor 594-conjugated goat anti-rabbit IgG (H+L) was purchased from Cell Signaling Technology (Danvers, MA, USA). Alexa Fluor 594-conjugated goat anti-mouse IgG (H+L) was purchased from Abcam (Cambridge, UK). The LD probes oil red O and Bodipy 493/503 were purchased from Beyotime (Shanghai, China) and GLPBIO (CA, USA). C75, TOFA, IBMX, palmitic acid (PA), atglistatin, and CAY10499 were purchased from GLPBIO (CA, USA). Triacsin C and isoproterenol were purchased from Abcam and MedChemExpress (USA).

### Experimental fish and sample collection.

Approximately 200 5-month-old grass carp with an average weight and length of 8 g and 12 cm, respectively, were used in this study. The fish were bred and cultivated at the GuanQiao Experimental Station, Institute of Hydrobiology, Chinese Academy of Sciences (CAS). Fish were fed commercial feed twice daily, and water was exchanged daily. If no abnormal symptoms were observed, fish were selected for the viral challenge experiment. Fish were infected with GCRV at a dose of 20 μL/g of body weight by intraperitoneal injection. Before and specific days (1, 3, 5, and 7 dpi) after injection, 15 fish were anesthetized and euthanized with MS-222 (100 mg/L), and their livers were removed and stored at −80°C for further analysis.

### Liver lipidomic analysis.

The liver samples were frozen immediately in liquid nitrogen and then preserved at −80°C. The samples were thawed slowly on ice, and 20 mg of each sample was homogenized in a 1-mL mixture (including methanol, methyl tert-butyl ether (MTBE), and internal standard mixture). After homogenization, the mixture was vortexed and then centrifuged for 10 min at 12,000 rpm and 4°C. A 200-μL volume of the upper organic layer was collected and evaporated using a vacuum concentrator. The dry extract was reconstituted using 200 μL of mobile phase B before liquid chromatography-tandem mass spectrometry (LC-MS/MS) analysis.

Lipidomic analysis by LC-MS/MS was performed by Metware Biotechnology Co., Ltd. (Wuhan, China) based on a previously described protocol but with slight modifications (52). Briefly, sample extracts were analyzed using an LC-ESI-MS/MS system (ultraperformance liquid chromatography [UPLC], ExionLC, AD, MS, triple quadrupole linear ion trap [QTRAP] system). The analytical conditions were as follows: UPLC, column, Thermo Accucore C30 (2.6 μm, 2.1 mm by 100 mm [inside diameter]); solvent system A, acetonitrile/water (60/40 [vol/vol], 0.1% formic acid, and 10 mmol/L of ammonium formate); solvent system B, acetonitrile/isopropanol (10/90 [vol/vol], 0.1% formic acid, and 10 mmol/L of ammonium formate); gradient program (A/B), 80:20 (vol/vol) at 0 min, 70:30 (vol/vol) at 2 min, 40:60 (vol/vol) at 4 min, 15:85 (vol/vol) at 9 min, 10:90 (vol/vol) at 14 min, 5:95 (vol/vol) at 15.5 min, 5:95 (vol/vol) at 17.3 min, 80:20 (vol/vol) at 17.3 min, and 80:20 (vol/vol) at 20 min; flow rate, 0.35 mL/min; temperature, 45°C; and injection volume, 2 μL. The effluent was alternatively connected to an electrospray ionization (ESI)-QTRAP-MS system.

Linear ion trap (LIT) and triple quadrupole (QQQ) scans were acquired on a QTRAP mass spectrometer (QTRAP LC-MS/MS system) equipped with an ESI TurboIonSpray interface, operating in positive and negative ion modes and controlled by Analyst 1.6.3 software (Sciex). The ESI source operation parameters were as follows: ion source, turbo spray; source temperature, 500°C; ion spray voltage (IS), 5,500 V (positive) and −4,500 V (negative); ion source gas 1 (GS1), gas 2 (GS2), and curtain gas (CUR), 45, 55, and 35 lb/in^2^, respectively; and collision gas (CAD), medium. Instrument tuning and mass calibration were performed using 10- and 100-μmol/L polypropylene glycol solutions in the QQQ and LIT modes, respectively. QQQ scans were acquired as multiple reaction monitoring (MRM) experiments with the collision gas (nitrogen) set to 5 lb/in^2^. Declustering potential (DP) and collision energy (CE) for individual MRM transitions were performed with further DP and CE optimization. A specific set of MRM transitions was monitored for each period according to the metabolites eluted within this period.

### Plasmid construction and transfection.

The plasmid pEGFP-N3 was reconstructed to express the red fluorescence protein mCherry. Briefly, open reading frame (ORF) sequence of mCherry was amplified from pmCherry-C1 (Table S2). The PCR product was digested with BamHI and NotI and then inserted into pEGFP-N3, which was treated with the same enzymes. The resulting plasmid was named pmCherry-N3, in which the enhanced green fluorescent protein (EGFP) ORF was replaced by mCherry.

10.1128/mbio.02297-22.9 TABLE S2 Primer sequences used in the study. Download Table S2, DOCX file, 0.02 MB. Copyright © 2022 He et al. 2022 He et al. https://creativecommons.org/licenses/by/4.0/ This content is distributed under the terms of the Creative Commons Attribution 4.0 International license.

To express the 12 GCRV-encoded proteins fused with EGFP or mCherry, the ORF sequences of these proteins were amplified from GCRV genomic dsRNA by RT-PCR. PCR products were subcloned into the pEGFP-N3 or pmCherry-N3 vector using the infusion method. The resulting plasmids were confirmed by DNA sequencing. Primers used for plasmid construction are listed in Table S2.

Transfection was performed as previously described (53), but with some modifications. GCO cells grown in glass-bottomed cell culture dishes were transfected with plasmids using the *Trans*IT-LTI transfection reagent (Mirus, USA) according to the manufacturer’s instructions. After 24 h posttransfection, cells were fixed with 4% paraformaldehyde, permeabilized with 0.2% Triton X-100, and stained with Hoechst 33342. Finally, the cells were mounted in 50% glycerol and observed under a confocal microscope (Leica, Germany).

### Immunofluorescence microscopy.

GCO cells grown in glass-bottomed cell culture dishes were mock infected or infected with GCRV at an MOI of 1 and harvested at 24 hpi. Cells were fixed with 4% paraformaldehyde for 30 min. Fixed cells were permeabilized with 0.2% Triton X-100 and then blocked in 10% normal goat serum at room temperature for 1 h. The cells were incubated with primary antibodies (rabbit anti-NS80, rabbit anti-VP5, or mouse anti-double-stranded RNA) diluted in 1% normal goat serum for 2 h, rinsed three times for 10 min each with phosphate-buffered saline (PBS) containing 1% normal goat serum, and then incubated with secondary antibodies (Alexa Fluor 594-conjugated goat anti-rabbit IgG or Alexa Fluor 594-conjugated goat anti-mouse IgG). Hoechst 33342 staining was used to visualize the nuclei. Finally, the cells were rinsed with PBS, mounted with 50% glycerol, and visualized using a confocal microscope (Leica, Germany).

### Transmission electron microscopy.

Transmission electron microscopy (TEM) experiments were performed as previously described (54), with some modifications. GCO cells were mock infected or infected with GCRV at an MOI of 1 and harvested at 48 hpi by centrifugation at 2,000 × *g* for 5 min. The pellets were prefixed with 2.5% glutaraldehyde for 24 h at 4°C, followed by postfixation with 1% osmium tetroxide (OsO_4_) for 2 h at 4°C. The samples were dehydrated stepwise in a graded series of ethanol and embedded in the epoxy resin Epon-812 overnight. The specimens were cut using a Leica DMIRB ultrathin microtome at a 70-nm thickness, double stained with uranyl acetate and lead citrate, and observed with an HC-1 80.0-kV Hitachi TEM system (Hitachi, Japan).

### Lipid droplet staining.

The formation of lipid droplets during GCRV infection *in vivo* was detected using oil red O staining. Liver samples from mock-infected or GCRV-infected grass carp were fixed in 4% paraformaldehyde overnight at 4°C. Following dehydration, the samples were embedded in HistoResin (Leica, Germany). Serial sections with a thickness of 4 mm were cut using a microtome (Leica) and dried on slides at 42°C overnight. The sections were then stained with fresh oil red O in a working solution (0.5% oil red O in isopropanol-water at 3:2) for 15 min. Then, the sections were washed twice with 60% isopropanol to remove any background. Finally, sections were washed with double-distilled water (ddH_2_O), mounted with 50% glycerol, and observed under a light microscope (Leica, Germany).

In addition, Bodipy 493/503 staining was performed to detect LD formation during GCRV infection *in vitro*. GCO cells grown in glass-bottomed cell culture dishes were mock infected or infected with GCRV at an MOI of 1 and harvested at 24 hpi. Cells were fixed with 4% paraformaldehyde for 30 min. The fixed cells were incubated with 5 μM Bodipy 493/503 for 15 min and then stained with Hoechst 33342. Images were acquired using a confocal microscope (Leica, Germany).

### RT-qPCR.

RT-qPCR was used to investigate the effects of the various compounds on the mRNA expression levels of viral genes (Table S2). Total RNA was isolated using the AG RNAex Pro reagent (Accurate Biology, China), and first-strand cDNA was obtained using a HiScript III first-strand cDNA synthesis kit (Vazyme, China). RT-qPCR was performed using a fluorescence quantitative PCR instrument (Bio-Rad, USA). Each reaction mixture contained 0.8 μL of forward and reverse primers (for each primer), 1 μL of cDNA template, 10 μL of 2× SYBR qPCR master mix (Vazyme, China), and 7.4 μL of ddH_2_O. Three replicates were performed for each sample, and the *β-actin* gene was used as an internal control for the normalization of gene expression. The program was as follows: 95°C for 10 s, 40 cycles of 95°C for 15 s, 56°C for 30 s, and 72°C for 30 s, and melt curve construction. The relative expression levels were calculated using the threshold cycle (2^−ΔΔ^*^CT^*) method (55). Data are presented as means ± standard deviations (SD) of three replicates.

### Plaque assay.

Plaque assay was performed to investigate the effects of the various compounds on progeny virus production. Cells were treated with different compounds and infected with GCRV. Supernatants were collected from differently treated cells at specific time points after GCRV infection and then used to infect fresh cells in 12-well plates. The cells were overlaid with a medium containing 0.7% melted soft agar. After 24 to 48 hpi, plaques formed, and the medium was removed. The cells were then fixed with 4% paraformaldehyde and stained with 1% crystal violet.

### Cell viability detection.

The CCK-8 detection kit (Beyotime, China) was used to investigate the effects of different compounds on cell viability, as described previously (7). Briefly, approximately 5 × 10^3^ GCO cells were seeded into 96-well plates. Cells were treated with compounds at different concentrations for 24 h. Then, 10 μL of CCK-8 solution was added to each well and incubated at 28°C for 4 h, and the absorbance at 450 nm was measured using a microplate reader (Bio-Rad, USA). The untreated cells were considered the positive control, while the wells containing no cells and only culture medium were used as blank controls. Data are presented as the means ± SD of three replicates.

### Statistical analysis.

All experiments were performed three times. One-way analysis of variance (ANOVA) and unpaired two-tailed Student’s *t* test were used to analyze statistical significance using SPSS 19 software. In figures, statistical significance is indicated by asterisks (*, *P* = 0.05 to 0.01; **, *P* = 0.01 to 0.001; ***, *P* ≤ 0.001).
