# Supplementary figures and images for: Grass Carp Reovirus Induces Formation of Lipid Droplets as Sites for Its Replication and Assembly
Source: mBio. 2022 Nov 29;13(6):e02297-22. doi: 10.1128/mbio.02297-22 (PMC9765412; doi:10.1128/mbio.02297-22)

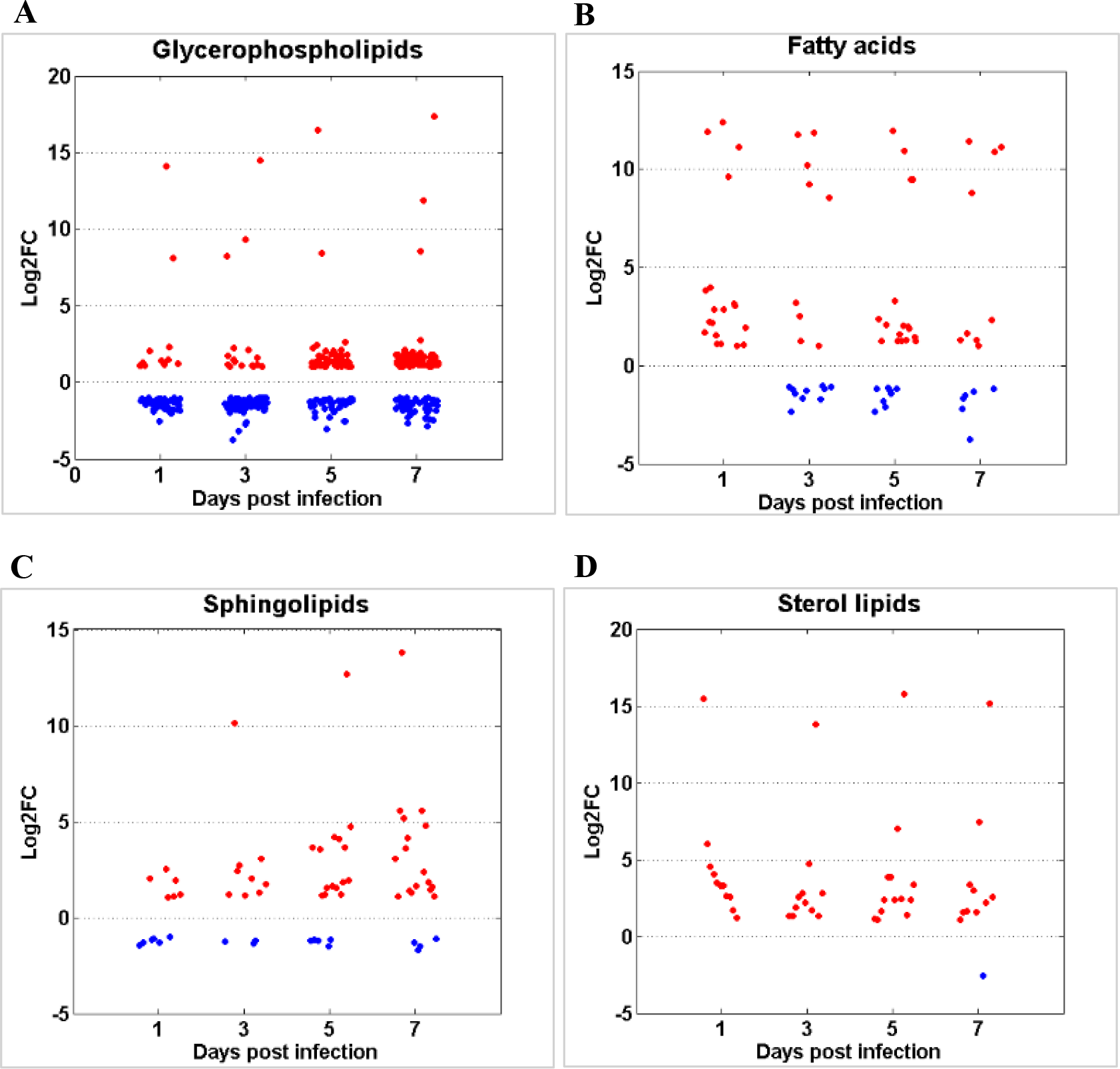

Supplement: FIG S1 [file mbio.02297-22-s0001.tif]

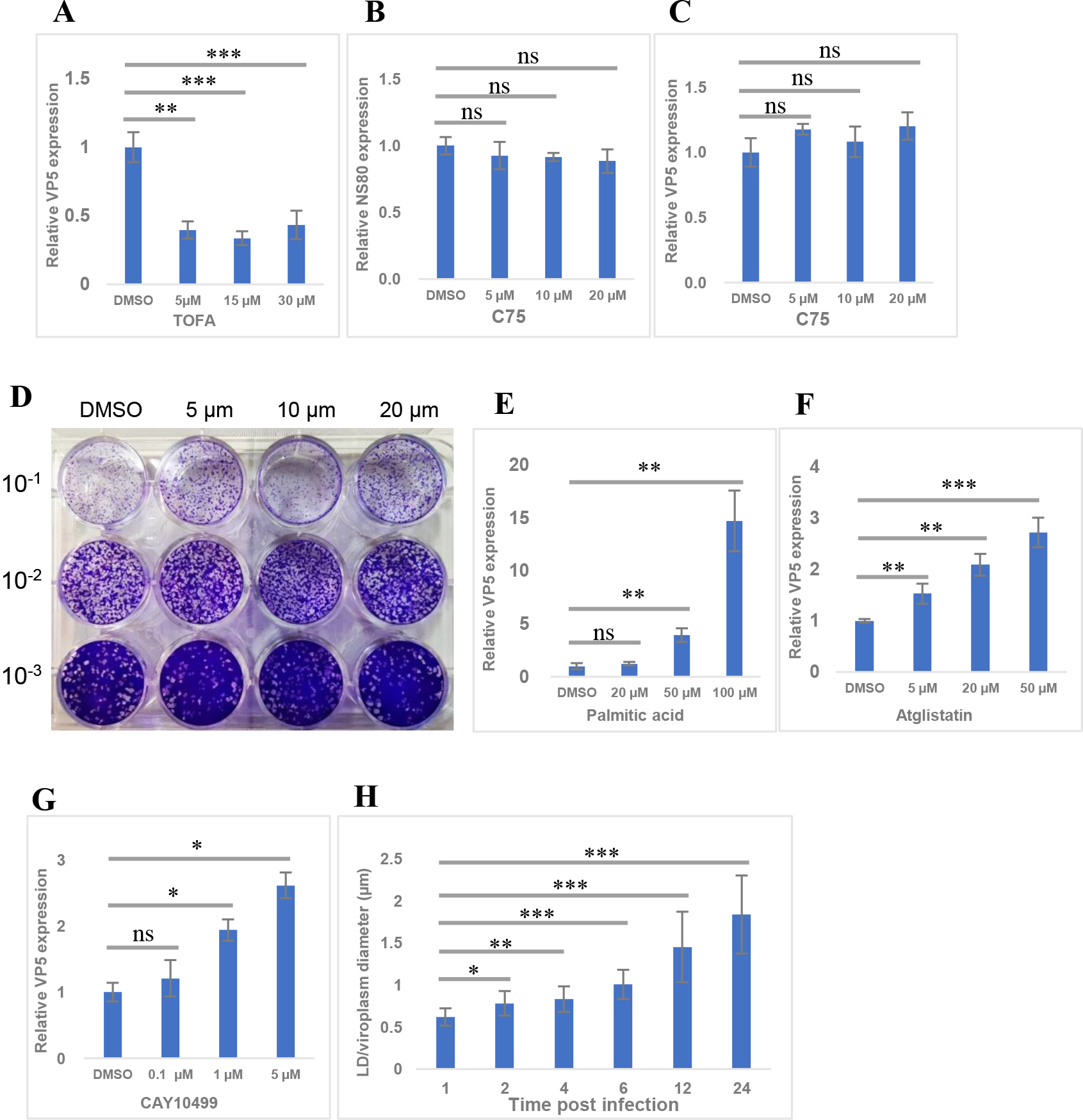

Supplement: FIG S2 [file mbio.02297-22-s0002.tif]

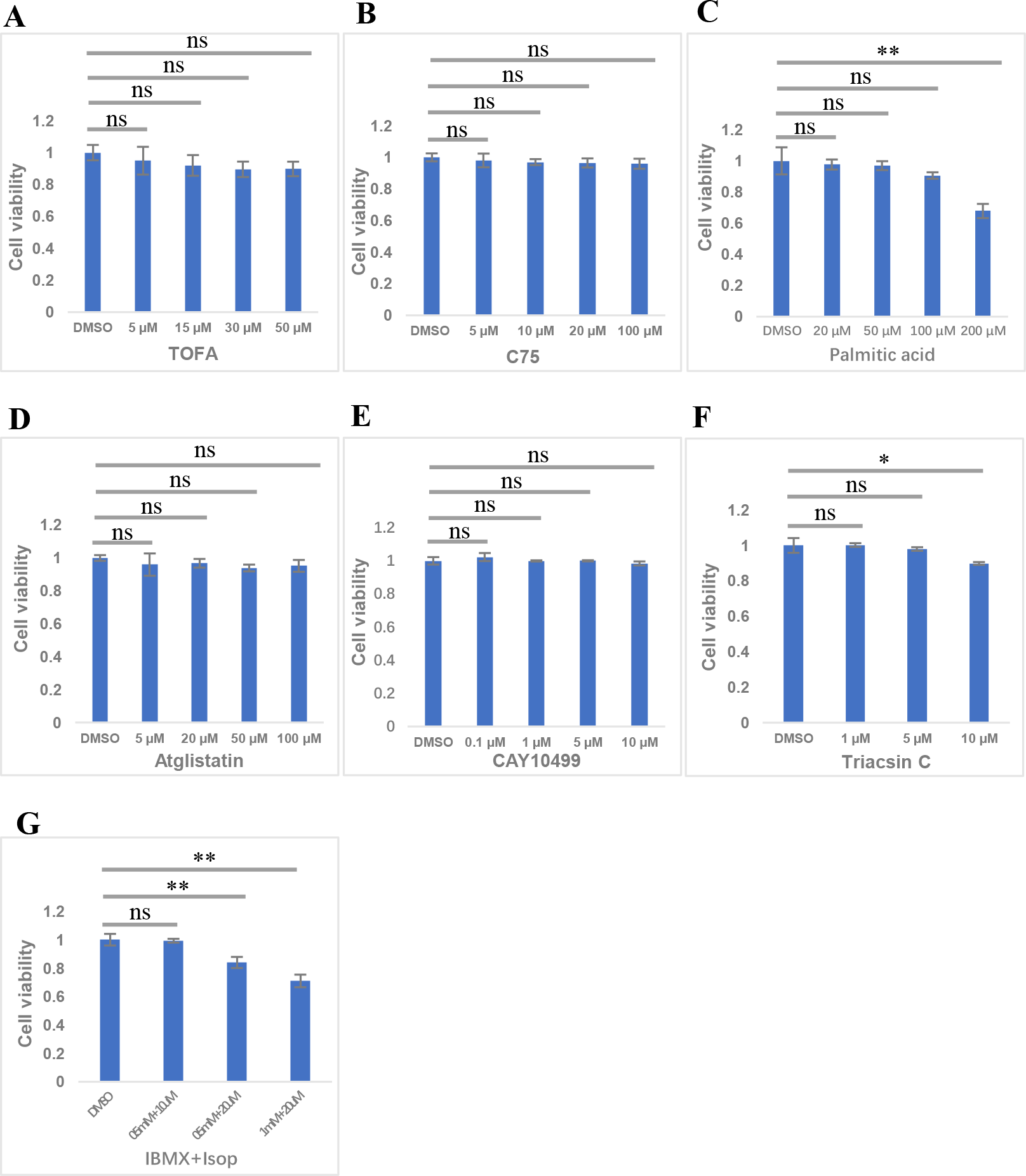

Supplement: FIG S3 [file mbio.02297-22-s0003.tif]

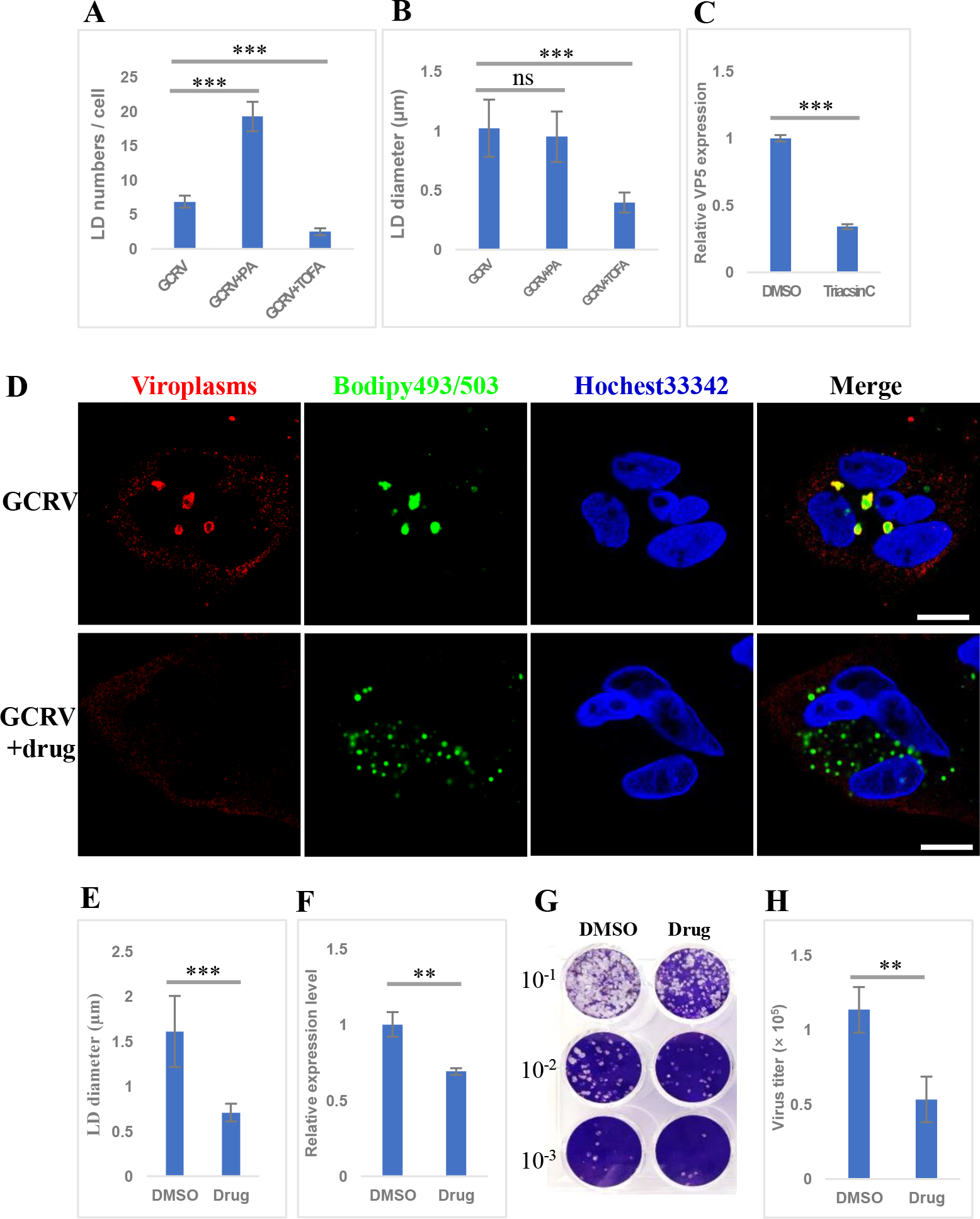

Supplement: FIG S4 [file mbio.02297-22-s0004.tif]

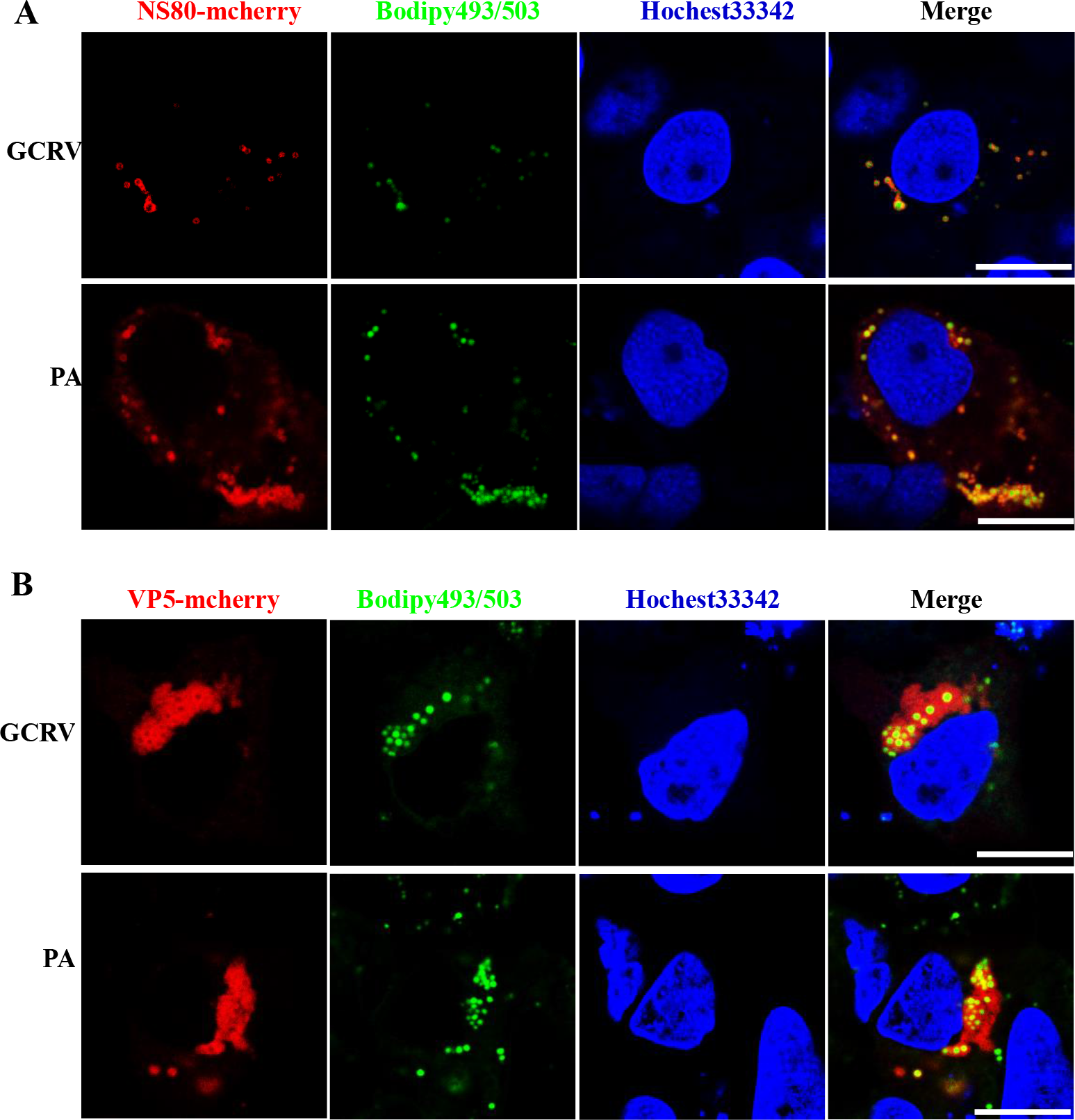

Supplement: FIG S6 [file mbio.02297-22-s0006.tif]
