# Supplementary material for: Grass Carp Reovirus Induces Formation of Lipid Droplets as Sites for Its Replication and Assembly
Source: mBio. 2022 Nov 29;13(6):e02297-22. doi: 10.1128/mbio.02297-22 (PMC9765412; doi:10.1128/mbio.02297-22)
Supplement: TABLE S2 [file mbio.02297-22-s0009.docx]

**Table S2 Primer sequences used in the study**

| **primers** | **Sequences (5’ to 3’ )** | **usage** |
| --- | --- | --- |
| mCherry-F | CCGGGATCCATCGCCACCATGGTGAGCAAGGG | Mcherry amplification |
| mCherry-R | AGTCGCGGCCGCTCTACTTGTACAGCTC |  |
| S1-F | TCAGATCTCGAGCTCAAGCTTCGGCCACCATGGCTGCGGTTTTCGGC | S1 (VP1) amplification |
| S1-R | GGATCCCGGGCCCGCGGTACCCTCAATCACGTATTCGTACACG |  |
| S2-F | TCAGATCTCGAGCTCAAGCTTCGGCCACCATGGAGGAATTGTTCAACGCC | S2 (VP2) amplification |
| S2-R | GGATCCCGGGCCCGCGGTACCAACATCACGCATCCAGACC |  |
| S3-F | TCAGATCTCGAGCTCAAGCTTCGGCCACCATGCCGCGCCGATCAGCCC | S3 (VP3) amplification |
| S3-R | GGATCCCGGGCCCGCGGTACCCGTCGCGCTGCGCACGTAC |  |
| S4-F | TCAGATCTCGAGCTCAAGCTTCGGCCACCATGGCACGCCGCATTACTTTG | S4 (NS80) amplification |
| S4-R | GGATCCCGGGCCCGCGGTACCCAGCAGCAGGGAGGCAGGG |  |
| S5-F | TCAGATCTCGAGCTCAAGCTTCGGCCACCATGATCACCATTGTGGTTATTCCA | S5 (VP4) amplification |
| S5-R | GGATCCCGGGCCCGCGGTACCAACCCCGGTCGAGGTGACAAC |  |
| S6-F | TCAGATCTCGAGCTCAAGCTTCGGCCACCATGTGGAACGTTCAAACCTCC | S6 (VP5) amplification |
| S6-R | GGATCCCGGGCCCGCGGTACCCTTGCCGGGCCACAAGTTC |  |
| S7-F1 | TCAGATCTCGAGCTCAAGCTTCGGCCACCATGCCTTGCCAGGATACTGTC | S7-1 (NS16) amplification |
| S7-R1 | GGATCCCGGGCCCGCGGTACCCTGAAACGCCTCAGGCGTGT |  |
| S7-F2 | TCAGATCTCGAGCTCAAGCTTCGGCCACCATGAACGCCGACCGCACCT | S7-2 (NS31) amplification |
| S7-R2 | GGATCCCGGGCCCGCGGTACCGCAACCATTGTCCATTAACTGG |  |
| S8-F | TCAGATCTCGAGCTCAAGCTTCGGCCACCATGGCACAGCGTCAGTTTTTC | S8 (VP6) amplification |
| S8-R | GGATCCCGGGCCCGCGGTACCGACGAACATCGCCTGCGCTA |  |
| S9-F | TCAGATCTCGAGCTCAAGCTTCGGCCACCATGGCACACACAGGCACCG | S9 (NS38) amplification |
| S9-R | GGATCCCGGGCCCGCGGTACCCATACCCCCGATCGGCAGG |  |
| S10-F | TCAGATCTCGAGCTCAAGCTTCGGCCACCATGCCACTTCACATGATTCCG | S10 (VP7) amplification |
| S10-R | GGATCCCGGGCCCGCGGTACCATCGGATGGCTCCACATGCAA |  |
| S11-F | TCAGATCTCGAGCTCAAGCTTCGGCCACCATGGCCACCGCAGGAATCA | S11 (NS25) amplification |
| S11-R | GGATCCCGGGCCCGCGGTACCTTCAAACACGCACGACATTGG |  |
| qNS80-F | GTCACTGCCCATTGCTTCGC | QPCR of *NS80* |
| qNS80-R | GCGGCACGGGATCTGTTGTA |  |
| qVP5-F | CCCGGAACAAGGCTCACCAT | QPCR of *VP5* |
| qVP5-R | GCGTGAGCAGTCTCCAGCTT |  |
| qactin-F | AGCCATCCTTCTTGGGTATG | QPCR of *actin* |
| qactin-R | GGTGGGGCGATGATCTTGAT |  |
